# Vaccination Education Deficits and Vaccine Hesitancy Among Healthcare Students in Japan: A Cross-Sectional Study

**DOI:** 10.3390/vaccines12121310

**Published:** 2024-11-22

**Authors:** Aya Saitoh, Hiromi Oku, Tomohiro Katsuta, Hajime Kamiya, Yoichi Ishikawa, Mayumi Takaku, Akihiko Saitoh

**Affiliations:** 1 Department of Nursing, Niigata University Graduate School of Health Sciences, Niigata 951-8518, Japan; 2 Department of Nursing, St. Luke’s International University Graduate School of Nursing Science, Tokyo 104-0044, Japan; 3 Department of Pediatrics, St. Marianna University Yokohama Seibu Hospital, Yokohama 241-0811, Japan; 4 Department of Public Health and Occupational Medicine, Mie University, Mie 514-8507, Japan; 5 Department of Pharmacy and Health Sciences, Meiji Pharmaceutical University, Tokyo 204-0004, Japan; 6 Department of Pediatrics, Niigata University Graduate School of Medical and Dental Sciences, Niigata 951-8510, Japan

**Keywords:** healthcare professional students, immunization education, Japan, vaccine hesitancy

## Abstract

Background/Objectives: Healthcare professionals’ (HCPs’) accurate knowledge of and positive attitudes toward immunization greatly influence society’s acceptance of it. Early and appropriate immunization education for HCP students is vital. This study aimed to understand current immunization education and vaccine hesitancy among medical, nursing, and pharmacy students in Japan. Methods: An anonymous self-administered online questionnaire was administered to final-year medical, nursing, and pharmacy students in Japan between 6 and 31 March 2023. Survey items assessed current immunization education, preparedness for clinical practice, immunization knowledge, and the degree of vaccine hesitancy. Results: Overall, 525 students (127 (24.2%) medical, 252 (48.0%) nursing, and 146 (27.8%) pharmacy) responded, of whom 39.8% raised concerns regarding new vaccine risks (24.4%,15.9%, and 23.3%, respectively; *p* = 0.22) and adverse effects (14.2%, 12.7%, and 17.1%, respectively; *p* = 0.57), including trust in government information (61.4%, 50/4%, and 56.8%, respectively; *p* = 0.337) or recommended vaccines (57.5%, 4.7%, and 43.8%, respectively; *p* = 0.113). Preparedness for future clinical practice varied significantly among schools, with medical students (54%) feeling more prepared compared to nursing (34.3%) and pharmacy students (39.1%) (*p* < 0.001). The average correct immunization knowledge rate was 59.9%, with significant differences between schools (medical 62.7%, nursing 57.6%, and pharmacy 59.6%; *p* < 0.001). There was no significant correlation between knowledge level and self-assessed preparedness (r = 0.066, *p* = 0.132). The HCP students wished to receive more immunization education and sought improvements in comprehensive knowledge, communication skills, and practice-based content. Conclusions: For Japan’s HCP students, the enhancement of immunization education focusing on hesitancy and risk–benefit communication is necessary.

## 1. Introduction

In recent years, vaccine hesitancy has emerged as a significant global health challenge. The World Health Organization (WHO) identified vaccine hesitancy among the top ten global health threats in 2019 [1]. This phenomenon, defined as the reluctance or refusal to vaccinate despite the availability of vaccines, is complex and context-specific, varying across time, place, and vaccines [1].

Japan presents a unique case in the global landscape of vaccine hesitancy. Despite its advanced healthcare system, Japan is known to have one of the lowest confidence levels in vaccines worldwide [2], particularly among the younger generation. This low confidence is rooted in a complex history of vaccine-related controversies and public health policies [3]. For instance, the suspension of the HPV vaccine recommendation in 2013 due to alleged adverse effects significantly impacted public trust in vaccines [4].

To address vaccine hesitancy and improve immunization rates, especially among young people, it is essential to advance their knowledge, attitudes, and beliefs toward immunization. This is where the concept of vaccine literacy becomes crucial [5]. Vaccine literacy, an extension of health literacy, refers to the degree to which individuals can obtain, process, and understand basic vaccine information and services needed to make appropriate vaccination decisions [6]. Enhancing vaccine literacy is increasingly recognized as a key strategy in combating vaccine hesitancy and promoting informed decision-making about vaccination [5].

Healthcare professionals (HCPs) play a pivotal role in this context. They are often the most trusted sources of information in the vaccination decision-making process. The knowledge, attitudes, and communication skills of HCPs regarding vaccination greatly influence their own vaccination rates, vaccine recommendations, and patients’ vaccination decisions [7,8]. However, several studies have revealed that some HCPs are themselves hesitant to vaccinate or recommend vaccines to their patients [9,10,11,12]. This hesitancy among HCPs is particularly concerning as it can significantly impact public confidence in vaccines.

The root cause of this hesitancy among HCPs could trace back to their formative years of education. Future HCPs, including students in medical, nursing, and pharmaceutical fields, are not immune to the influences of vaccine hesitancy. Studies in Europe and the United States during the COVID-19 pandemic indicated that 10–20% of medical and pharmaceutical students showed vaccine hesitancy [13,14,15]. Similarly, research in Turkey found that 11.8% of midwifery and nursing students expressed hesitation regarding infantile immunization.

Currently, data available on the education of HCP students in the field of immunization are insufficient, particularly in the Japanese context. The current state of vaccine hesitancy among HCP students, the reality of vaccine education for these students, their knowledge about vaccines and readiness for practice after graduation, and the differences or commonalities based on their field of specialization remain largely unknown. Evaluating these aspects is crucial for developing more effective educational programs that can enhance vaccine literacy among future HCPs and, by extension, the general public.

This study aims to address these knowledge gaps by revealing the following key issues among medical, nursing, and pharmacy students in Japan: (1) the current status of immunization education; (2) their preparedness for future clinical practice related to vaccination; (3) the prevalence and nature of vaccine hesitancy; and (4) their knowledge about immunization. By examining these factors across different healthcare disciplines, we aim to provide a comprehensive picture of the challenges and opportunities in educating future HCPs about vaccines and vaccination in Japan.

## 2. Materials and Methods

### 2.1. Design

This cross-sectional descriptive study was conducted using an anonymous self-administered web survey.

### 2.2. Setting and Participants

The study targeted final-year students, that is, sixth-year medical students, sixth-year pharmacy students, and fourth-year nursing students, to elucidate the existing pattern of vaccination education and the prevalence of vaccine hesitancy among HCP students in Japan.

The study participants were recruited from among the registered members of Rakuten Insight, Inc., a Japanese internet research company. The company distributed the questionnaire developed by researchers to medical, pharmacy, and nursing students residing in Japan via email using the designated website. Prior to accessing the survey, participants were provided with a detailed information sheet explaining the purpose of the study, the voluntary nature of participation, the types of questions they would be asked, and how their data would be used and protected. This information was presented on the initial screen of the survey platform.

Participants were explicitly informed that by proceeding with the survey questions, they were providing their informed consent to participate in the study and for their anonymized data to be used for research purposes. The information sheet also clearly stated that participants could withdraw from the study at any time by closing their browser window, and that partial responses would not be saved or used. Response to the survey was considered an agreement to cooperate.

### 2.3. Data Collection

The study data were collected between 6 and 31 March 2023. The survey items were as follows.

#### 2.3.1. Perceived Preparedness for Clinical Practice After Graduation

The study participants were asked to list all their course subjects, including content related to vaccination education. Perceived preparedness was assessed based on items from a study conducted on French medical students [16] modified to fit the Japanese context. A few items related to the epidemiologic characteristics of vaccine-preventable diseases that were deemed difficult to answer by experts in each field, as well as case-based questions, were excluded. Additionally, items with differences in the immunization system between France and Japan were revised and utilized.

The perceived level of preparedness was assessed using 22 items across five major objectives: (1) the immunological principles of vaccines and vaccine composition, (2) Japan’s immunization policies, (3) communication with patients, (4) the practical aspects of immunization, and (5) useful information sources for patients and HCPs. Participants rated their preparedness for each item using a 5-point Likert scale (1 = not prepared at all; 5 = extremely prepared), consistent with the scale used in the original study by Kernéis et al. [15]. For assessing training status on vaccination-related topics, participants responded to the question “Did your professional program (medicine, pharmacy, nursing) include appropriate training and education on the topics listed below?” using the same 5-point scale (1 = strongly disagree; 5 = strongly agree).

#### 2.3.2. Knowledge

Basic immunization-related knowledge was measured using multiple-choice questions (Supplementary Table S1). Knowledge scores were calculated based on participants’ responses, with 1 point for a correct answer and 0 points for an incorrect answer. The total knowledge score (maximum of 69 points) was determined by summing the number of correctly answered questions. Self-reported knowledge of pediatric immunization was assessed using a 19-item Likert scale scored as 1 (I do not know), 2 (I know a little), and 3 (I know). The total knowledge score (maximum of 69 points) was determined by summing the number of correctly answered questions.

#### 2.3.3. Adult Vaccine Hesitancy Scale

The adult Vaccine Hesitancy Scale (aVHS) comprises 10 items developed by the World Health Organization Vaccine Hesitancy Advisory Group [17] and evaluated on a 5-point Likert scale ranging from 1 (least hesitant) to 5 (most hesitant). The aVHS is a valid and reliable tool for assessing participants’ attitudes toward immunization against vaccine-preventable diseases [17]. A high score on this scale indicates higher vaccine hesitancy. The original text was translated from English to Japanese and its accuracy was confirmed through back translation by three proficient English translators. The questionnaire was subsequently tested using a pilot study.

### 2.4. Statistical Analysis

For our sample size calculation, we used the prevalence of vaccine hesitancy among HCPs as a reference, given the limited data on healthcare students specifically. Based on a systematic review by Paterson et al. [18], which reported vaccine hesitancy rates among HCPs ranging from 2% to 73%, we used the median value of approximately 30% as a conservative estimate for our calculation. Assuming a power of 0.80 and an alpha level of 0.05, and accounting for a 20% dropout rate, we determined a minimum sample size of 167 participants per group (medical, nursing, and pharmacy students).

Descriptive statistics were calculated for various course subjects related to vaccine education. For analyzing preparedness for clinical practice after graduation, knowledge, and aVHS scores, the results were presented as n (%) or a mean (SD). To compare the three groups (medical, nursing, and pharmacy students), chi-square tests were used for categorical variables and the Kruskal–Wallis test for quantitative variables. Similarly, variables indicating the level of preparedness were categorized into very well prepared and adequately prepared for sufficient preparedness and completely unprepared and inadequately prepared for insufficient preparedness, with a third category of do not know, for analysis.

We evaluated the relationship between knowledge level and clinical readiness (assessed on a 5-point Likert scale) by Spearman’s rank correlation analysis.

Data were analyzed using SPSS version 24.0 (IBM Corp., Armonk, NY, USA). Two-tailed *p*-values were used for all statistical tests. We set the significance level (α) at 0.05 for all statistical tests conducted in this study.

## 3. Results

### 3.1. Basic Characteristics

The survey was distributed to a total of 1200 students (400 each from medical, nursing, and pharmacy schools). Responses were obtained from a total of 525 participants, resulting in an overall response rate of 43.8%. The breakdown of respondents included 127 (24.2%) medical students, 252 (48.0%) nursing students, and 146 (27.8%) pharmacy students. The response rates for each group were as follows: 31.8% for the medical students (127/400), 63.0% for the nursing students (252/400), and 36.5% for the pharmacy students (146/400). In total, 43.6% of institutions were affiliated with national or public universities, and 56.4% were affiliated with private universities (Table 1).

### 3.2. Current Status of Immunization Education

#### 3.2.1. Courses with Content Related to Immunization

The study courses that included content related to immunization covered a wide range of subjects, including basic medical sciences, ethics, and management. Medical students listed subjects related to infectious diseases, such as infection prevention (34.6%) and courses related to immunology (29.1%). Conversely, nursing students listed pediatric nursing (19.0%) and maternal nursing (12.3%), whereas pharmacy students listed pharmacology (23.3%) and hygiene (23.3%) (Supplementary Table S2).

#### 3.2.2. Desire for Improvement in Immunization Education for Healthcare Profession Students

In response to the question on how immunization education for HCP students could be improved, we identified 28 subcategories, grouped into four main categories: (1) *acquiring comprehensive knowledge* (including learning about vaccines’ pros and cons, adverse reactions, and effectiveness); (2) *gaining communication skills* (for addressing vaccine hesitancy); (3) *content aligned with practical application* (such as field visits); and (4) *motivation for learning* (strategies to stimulate active thinking and curiosity among students). The number of participants who answered *nothing in particular* was 124 (23.6%) (Supplementary Table S3).

### 3.3. Perceived Preparedness for Future Clinical Practice

The average percentage of participants who answered *well prepared* to all items was low, being 54.0% among medical students versus 34.3% among nursing students and 39.1% among pharmacy students (*p* < 0.001) (Table 2).

In all three types of schools, themes such as communication methods for addressing vaccine hesitancy (47.2% for medical, 37.3% for nursing, and 25.3% for pharmacy students) and communication techniques (risk–benefit communication) (53.5% for medical, 43.7% for nursing, and 40.4% for pharmacy students.) featured the lowest rates of participants with appropriate training education (Table 3).

The proportion of participants who wished to receive more immunization education was high in all three schools: 68.5% for medical students, 70.2% for nursing students, and 66.4% for pharmacy students. No significant differences were observed among schools (*p* = 0.73).

### 3.4. The Adult Vaccine Hesitancy Scale (aVHS)

The majority of healthcare students demonstrated a high level of recognition regarding the importance, effectiveness, and public health necessity of vaccines. The proportion of students who considered vaccines important was 81.1% in medical schools, 73.8% in nursing schools, and 71.9% in pharmacy schools (*p* = 0.016). Similarly, high recognition was observed for vaccine effectiveness (81.1% in medical, 68.3% in nursing, and 73.3% in pharmacy schools; *p* = 0.004).

However, 15-25% of students perceived new vaccines as more dangerous, with medical (24.4%) and pharmacy (23.3%) students showing slightly higher proportions compared to nursing students (15.9%) (*p* = 0.22). Similar trends were observed regarding concerns about adverse reactions, with 14.2% of medical students, 12.7% of nursing students, and 17.1% of pharmacy students (*p* = 0.576).

**Figure 1:**
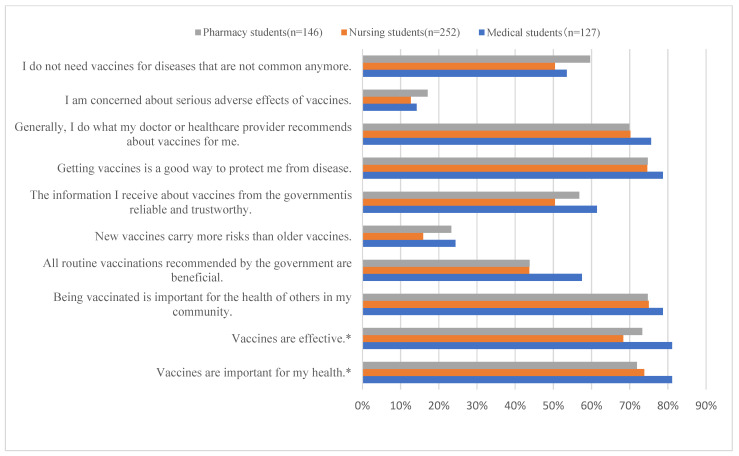
Comparison of percentage of positive responses (strongly agree/agree) on the adult Vaccine Avoidance Scale (aVHS) among health sciences departments (* *p*-value < 0.05).

Trust in vaccine information provided by the government and agreement with recommended vaccines remained at approximately half of the total sample. Among medical students, 57.5% trusted government information and agreed with recommended vaccines, compared to 43.7% of nursing students and 43.8% of pharmacy students. No difference was observed between the schools in this regard (*p* = 0.113) (Figure 1).

### 3.5. Knowledge

The average correct response rates for all knowledge questions were low: 62.7% for medical, 57.6% for nursing, and 59.6% for pharmacy students. Basic knowledge about vaccinations was the area with the lowest proportion of correct answers for all schools, with 48.7% of medical, 38.8% of nursing, and 44.1% of pharmacy students providing correct responses (Table 4). There was no significant correlation between the knowledge level and self-assessed preparedness (r = 0.066; *p* = 0.132) (Table 4).

## 4. Discussion

This study evaluated the preparedness of HCP students in Japan for immunization, with a focus on vaccine hesitancy and risk–benefit communication. The results showed that, while medical students had an advantage over nursing and pharmacy students in preparedness for clinical practice after graduation, overall immunization education was inadequate across all three faculties. A lack of education on communication techniques for addressing vaccine hesitancy was particularly evident.

These results offer important insights into HCP students’ preparedness for postgraduate clinical practice and related education. Specifically, the finding that medical students reported higher preparedness for clinical practice compared to nursing and pharmacy students denotes that the current medical curriculum may be more effective in equipping students with necessary skills and knowledge. This highlights the need for similar improvements in the curricula of nursing and pharmacy schools to ensure that all HCP students are adequately prepared for their future roles. In a previous study, programs in nursing and pharmacy schools had a lower proportion of curriculum content with specific learning objectives and formal assessments than programs in medical schools [18]. It is conceivable that the same situation exists in Japan; therefore, these differences in the three faculties are attributed to variations in educational curricula, teaching methods, and students’ access to educational resources.

Regarding themes in which education and training were perceived as lacking, all the study participants generally believed that they had received sufficient education and training on the efficacy of vaccines and vaccine-preventable diseases. However, a lack of education was observed regarding communication for vaccine hesitancy and risk–benefit communication. Previous studies have reported similar findings regarding the preparedness of HCP students. For instance, a study in France found that 34% of final-year medical students believed they were unprepared to handle vaccine hesitancy scenarios [15]. Consistent with these findings, our study shows that more than half of the Japanese medical students also believed that they were inadequately trained in this area. However, our study extends these findings by including nursing and pharmacy students, revealing that only 37.3% and 25.3% of these students, respectively, received sufficient training in addressing vaccine hesitancy. This suggests a broader issue across different HCP disciplines that needs to be addressed. Effective communication regarding vaccine safety is crucial and the development of educational programs focusing on these themes is imperative for all three faculties.

We also found that students from all three faculties were not satisfied with the immunization education they received, indicating the need for a more comprehensive curriculum. It can be speculated that students, while feeling that they have not been adequately taught, may not be able to precisely indicate what they want to be instructed on. Similarly, a Canadian study found that 85% of medical students and 87% of nursing students expressed the need for more training in immunization [19]. Therefore, the development of a comprehensive immunization education program is necessary for all HCP schools. This area may be of interest for interprofessional education considering that each HCP has their own role in providing vaccines for individuals.

The majority of students, regardless of faculties, responded positively to the importance of vaccines for both their health and that of the community and the effectiveness of vaccines. This suggests that the HCP students were aware of the importance and effectiveness of immunization and valued their contribution to both personal and community health. However, it is important to note that 10–25% of students across all three faculties expressed concerns about new vaccines carrying more risks and displayed concerns about adverse reactions.

These findings align with recent studies on vaccine hesitancy in Japan. For instance, a recent study found that 10.9% of medical students did not complete the second dose of the COVID-19 vaccine, indicating some level of vaccine hesitancy among healthcare students in Japan [20]. Similarly, a previous study reported that the proportion of COVID-19 vaccine hesitancy among the general Japanese population was 11.3%, with higher rates among younger respondents [21]. These studies highlight the persistent challenge of vaccine hesitancy in Japan, even among those training to become healthcare professionals.

Our results showed that 10–25% of students expressed concerns about new vaccines, which align with recent international studies. For instance, a 2022 study found that 23% of medical students in Greece were hesitant about COVID-19 vaccines [22]. Similarly, research from Italy reported vaccine hesitancy rates of 13.9% among Italian healthcare students [23]. These comparisons suggest that vaccine hesitancy among healthcare students is a global phenomenon, not limited to Japan. In a previous French study including medicine, midwifery, physiotherapy, nursing, and other health-related fields, 44.5% of the participants were hesitant about the new COVID-19 vaccine [24]. Medical students were less hesitant than students from other schools, while nursing students were five times as hesitant as medical students. The lower levels of hesitancy in our study may reflect cultural differences or the impact of Japan’s specific historical context with vaccines, as explored by Okubo et al. [25].

Beliefs about new vaccines may be associated with uncertainties regarding vaccine safety and information accuracy. Additionally, the lack of significant differences among all three faculties indicates a common trend in students’ beliefs and concerns about vaccines, irrespective of faculties. This consistency across disciplines suggests that vaccine hesitancy is a broader issue in Japanese society, not limited to specific healthcare fields, as also noted by Nomura et al. [26]. The lack of significant differences in vaccine hesitancy across medical, nursing, and pharmacy students suggests that factors beyond formal education play a crucial role in shaping attitudes. Recent research has emphasized the importance of addressing vaccine hesitancy through multiple channels, including peer influence and social norms [27]. Recent study has also shown that exposure to vaccine-related conspiracy theories significantly impacts healthcare students’ attitudes towards vaccination [28]. This underscores the need for educational programs that not only provide factual information but also equip students with critical thinking skills to evaluate vaccine-related claims.

Since 2011, the Japanese government has issued a directive to implement educational programs on pharmaceutical harms, including vaccines, starting from junior high school. One of the reasons why is that the harmful effects of vaccines were related to the fact that there have been lawsuits against the government related to mumps and human papillomavirus vaccines [29]. However, descriptions related to the benefits of vaccines are lower. This study clarifies that students’ confidence in government information is generally low, particularly regarding the efficacy of vaccines recommended by the government. This suggests that, despite the government’s crucial role in providing vaccine information, some students harbored doubts about such information. This mistrust may not be solely attributable to vaccine hesitancy or concerns about the adverse effects of vaccines but rather from a broader mistrust of the entities advocating immunization [30]. As early education may impact this mistrust, it is necessary to consider various ways to address these beliefs and concerns and emphasize the importance of providing evidence-based information. There is a need for improving education and communication strategies for addressing concerns about new vaccines and building trust in information provided by the government. Further research is needed to understand how differences in educational approaches among faculties affect students’ beliefs. The influence of the internet and social media on vaccine attitudes cannot be overlooked, especially in the COVID-19 era. Recent research has highlighted that social media platforms have become primary sources of vaccine-related information for young adults in Japan [31]. This trend presents both challenges and opportunities for addressing vaccine hesitancy. While social media can rapidly spread misinformation, it also offers a platform for targeted educational interventions.

Understanding students’ awareness and beliefs and providing appropriate information are pivotal to combat vaccine hesitancy, which is rooted in historical and cultural factors [32] and poses a global challenge; however, methods to effectively contend this misgiving are limited. Thus, it is important to consistently demonstrate the effectiveness of HCP education. Traditional undergraduate medical education typically covers the legal framework of immunization, clinical information, immunization schedules, and proper administration techniques. However, educational institutions often exercise significant discretion in teaching these topics, resulting in limited opportunities for students to understand vaccine hesitancy systematically and comprehensively and develop communication skills. The findings of this study further emphasize the need for comprehensive instruction, as evidenced by the smaller number of classes dedicated to immunization, the reported insufficient knowledge among HCP students, and the desire for comprehensive courses encompassing communication skills, including specific approaches to dealing with patients’ vaccine concerns. Currently, HCPs have inadequate opportunities to acquire the practical knowledge and skills necessary to effectively navigate and address vaccine hesitancy.

Motivational interview training that is specific to immunization is easily adaptable to HCP in the immunization field and can promote immunization and discourage parental vaccine hesitancy [33]. Training in immunization education that is based on motivational interviewing techniques should be enhanced in basic HCP education.

On the question of administering vaccines when an infant is two months old, a greater number of medical students responded correctly than nursing and pharmacy students. This indicates that medical students excelled in their knowledge of specific vaccine schedules. Such knowledge is crucial for future clinical practice, and it is important to impart it to nursing and pharmacy students. However, less than half of the participating students answered questions related to basic knowledge about vaccination correctly, suggesting that education on basic knowledge about vaccines can be improved in all three faculties. In contrast, the results showed little to no correlation between knowledge level and self-assessed preparedness. This finding suggests that the acquisition of knowledge does not necessarily translate into confidence in clinical preparedness, indicating that practical skills and experience may have a greater influence on perceived readiness for clinical practice. A previous study indicated that extracurricular activities were highly effective in increasing immunization knowledge [34]. Further investigation into the format and types of educational methods, including extracurricular activities, may be necessary.

Future research should focus on evaluating the effectiveness of different educational approaches in improving HCP students’ preparedness for addressing vaccine hesitancy. Specifically, studies could investigate the impact of incorporating motivational interviewing techniques into the curriculum, as this is considered effective in other contexts [2]. Additionally, research should explore ways to enhance trust in vaccine information provided by the government, as this was identified as a significant concern among students. Longitudinal studies are also needed to assess the long-term impact of improved immunization education on HCP students’ clinical practice and patient outcomes.

## 5. Limitations

This study has several limitations. First, data were collected through an internet survey, potentially introducing a selection bias, as students with a particular interest in immunization may have been more likely to participate in the survey. Second, the study’s cross-sectional design limits the ability to draw causal inferences. Third, the questionnaire used in this study was not validated, which may affect the reliability and validity of our findings. Future studies should consider using or developing validated instruments to assess vaccine hesitancy and related factors among healthcare students.

Fourth, there was a gender imbalance in our sample. This gender bias may limit the generalizability of our findings and could potentially skew the results, particularly if there are significant gender differences in attitudes towards vaccination

Fifth, we collected basic demographic data (gender, age, type of university, and residential area) but did not include additional variables such as religion/spirituality, political orientation, or health status. Previous research has suggested that these factors may influence attitudes toward vaccination [35,36,37]. Including these variables might have allowed for a more comprehensive understanding of the factors influencing vaccine hesitancy among healthcare students in Japan. Additionally, the practical aspects of immunization education for pharmacy students in Japan are limited compared to other countries, as pharmacists are not authorized to administer vaccines. These limitations should be considered when interpreting the results, and future research must aim to address these issues by using more representative samples and longitudinal designs.

## 6. Conclusions

This study proposes directions for improving immunization education among Japanese HCP students. The low preparedness of HCP students highlights the need for focusing on education related to vaccine hesitancy and risk–benefit communication. Furthermore, HCP students’ knowledge differs among schools. Thus, educational programs should be designed based on the differences in knowledge among schools and specific areas of improvement. We strongly believe that providing basic knowledge and accurate information on immunization to HCP students will contribute toward improving future public health programs.

## Figures and Tables

**Table 1:** Sociodemographic characteristics of the study population.

Demographics		*n*	(%)
Types of schools			
	Medical students	127	(24.2)
	Nursing students	252	(48.0)
	Pharmacy students	146	(27.8)
Gender			
	Male	121	(23.0)
	Female	404	(77.0)
Age			
	≤24	471	(89.7)
	25–29	45	(8.6)
	30≤	9	(1.7)
Types of universities			
	National/public universities	229	(43.6)
	Private universities	296	(56.4)
Residential area			
	Tokyo metropolitan area	148	(28.2)
	Other area	377	(71.8)
Total		525	

**Table 2:** Comparison of clinical preparedness among healthcare profession students.

Q. How Well Do You Feel Prepared to Handle Vaccination in Future Clinical Settings?			Medical Students		Nursing Students		Pharmacy Students		*p* Value
			*n*	%	*n*	%	*n*	%	
**1**									
Immunology, Vaccines									
1	Explain to patients how immunity is acquired through vaccination	Poorly prepared	25	19.70%	76	30.20%	37	25.30%	<0.001
		Well prepared	74	58.30%	80	31.70%	65	44.50%	
		Don’t know	28	22.00%	96	38.10%	44	30.10%	
2	Explain to the patients the importance of collective immunity through vaccination to protect the community	Poorly prepared	25	19.70%	62	24.60%	32	21.90%	0.283
		Well prepared	68	53.50%	105	41.70%	65	44.50%	
		Don’t know	34	26.80%	85	33.70%	49	33.60%	
3	Explain to patients the difference between live and inactivated vaccines in the immunization schedule	Poorly prepared	25	19.70%	81	32.10%	27	18.50%	<0.001
		Well prepared	77	60.60%	97	38.50%	81	55.50%	
		Don’t know	25	19.70%	74	29.40%	38	26.00%	
Vaccination policy									
4	How to advise patients about the current immunization schedule	Poorly prepared	24	18.90%	73	29.00%	37	25.30%	0.019
		Well prepared	68	53.50%	92	36.50%	55	37.70%	
		Don’t know	35	27.60%	87	34.50%	54	37.00%	
5	How to advise travelers about recommended vaccines according to their destination	Poorly prepared	28	22.00%	91	36.10%	48	32.90%	0.001
		Well prepared	65	51.20%	81	32.10%	44	30.10%	
		Don’t know	34	26.80%	80	31.70%	54	37.00%	
6	Explain the importance of vaccination to healthcare professionals	Poorly prepared	25	19.70%	64	25.40%	27	18.50%	<0.001
		Well prepared	70	55.10%	84	33.30%	69	47.30%	
		Don’t know	32	25.20%	104	41.30%	50	34.20%	
7	Listing vaccines with low vaccination coverage in Japan	Poorly prepared	43	33.90%	127	50.40%	68	46.60%	0.028
		Well prepared	45	35.40%	57	22.60%	38	26.00%	
		Don’t know	39	30.70%	68	27.00%	40	27.40%	
8	Explanation of the difference between routine and voluntary vaccinations	Poorly prepared	16	12.60%	51	20.20%	22	15.10%	0.261
		Well prepared	82	64.60%	137	54.40%	85	58.20%	
		Don’t know	29	22.80%	64	25.40%	39	26.70%	
Communication									
9	Explanation of typical complications of vaccine-preventable diseases	Poorly prepared	23	18.10%	82	32.50%	41	28.10%	0.002
		Well prepared	69	54.30%	85	33.70%	54	37.00%	
		Don’t know	35	27.60%	85	33.70%	51	34.90%	
10	Answers to questions from patients about potential risks of vaccines	Poorly prepared	24	18.90%	87	34.50%	36	24.70%	<0.001
		Well prepared	62	48.80%	64	25.40%	52	35.60%	
		Don’t know	41	32.30%	101	40.10%	58	39.70%	
11	How to communicate with patients who are hesitant to receive vaccines (patients who have concerns about vaccine safety or efficacy)	Poorly prepared	26	20.50%	79	31.30%	45	30.80%	0.026
		Well prepared	58	45.70%	81	32.10%	42	28.80%	
		Don’t know	43	33.90%	92	36.50%	59	40.40%	
12	How to deal with patients who refuse to be vaccinated	Poorly prepared	32	25.20%	90	35.70%	53	36.30%	0.124
		Well prepared	50	39.40%	77	30.60%	39	26.70%	
		Don’t know	45	35.40%	85	33.70%	54	37.00%	
13	Explaining to patients about the benefits (effectiveness and necessity) of vaccination	Poorly prepared	16	12.60%	70	27.80%	22	15.10%	<0.001
		Well prepared	85	66.90%	96	38.10%	75	51.40%	
		Don’t know	26	20.50%	86	34.10%	49	33.60%	
14	Explaining to patients about adverse reactions associated with vaccination	Poorly prepared	15	11.80%	70	27.80%	33	22.60%	<0.001
		Well prepared	76	59.80%	88	34.90%	66	45.20%	
		Don’t know	36	28.30%	94	37.30%	47	32.20%	
**2**									
Practical skills									
15	Listing of items to be confirmed with patients prior to vaccination (precautions, contraindications, situations requiring postponement of vaccination)	Poorly prepared	20	15.70%	65	25.80%	33	22.60%	0.002 *
		Well prepared	80	63.00%	104	41.30%	64	43.80%	
		Don’t know	27	21.30%	83	32.90%	49	33.60%	
16	Procedures for administering vaccinations to patients (different vaccination methods for different vaccines, selection of vaccination sites according to age, post-vaccination monitoring)	Poorly prepared	21	16.50%	76	30.20%	41	28.10%	<0.001 *
		Well prepared	74	58.30%	87	34.50%	47	32.20%	
		Don’t know	32	25.20%	89	35.30%	58	39.70%	
17	Listing of items to be noted after vaccination	Poorly prepared	28	22.00%	85	33.70%	39	26.70%	0.004 *
		Well prepared	62	48.80%	77	30.60%	48	32.90%	
		Don’t know	37	29.10%	90	35.70%	59	40.40%	
18	Dealing with anaphylactic reactions associated with vaccination (behavior)	Poorly prepared	22	17.30%	77	30.60%	37	25.30%	<0.001 *
		Well prepared	79	62.20%	93	36.90%	61	41.80%	
		Don’t know	26	20.50%	82	32.50%	48	32.90%	
19	Explanation to patients about possible events after vaccination and measures to be taken in case of adverse reactions	Poorly prepared	30	23.60%	68	27.00%	24	16.40%	<0.001 *
		Well prepared	72	56.70%	90	35.70%	60	41.10%	
		Don’t know	25	19.70%	94	37.30%	62	42.50%	
Sources of information									
20	Searching for reliable sources of information about vaccines for healthcare professionals (vaccination schedules, efficacy and safety of various vaccines, etc.)	Poorly prepared	20	15.70%	76	30.20%	46	31.50%	<0.001 *
		Well prepared	64	50.40%	75	29.80%	46	31.50%	
		Don’t know	43	33.90%	101	40.10%	54	37.00%	
21	Search for reliable resources and information about vaccines for patients.	Poorly prepared	21	16.50%	70	27.80%	32	21.90%	0.001 *
		Well prepared	71	55.90%	85	33.70%	57	39.00%	
		Don’t know	35	27.60%	97	38.50%	57	39.00%	
Other									
22	Overall, how prepared do you feel you are to handle immunizations in your clinical practice in the future?	Poorly prepared	30	23.60%	88	34.90%	54	37.00%	0.003 *
		Well prepared	58	45.70%	67	26.60%	44	30.10%	
		Don’t know	39	30.70%	97	38.50%	48	32.90%	

Five-point Likert scale as answer choices, ranging from 1 (not prepared at all) to 5 (very well prepared). * *p*-value < 0.05.

**Table 3:** Percentage of students who answered that healthcare profession schools provide adequate immunization training and education-related topics.

	Medical Schools (*n* = 127)		Nursing Schools (*n* = 252)		Pharmacy Schools (*n* = 146)		*p*-Value
	*n*	%	*n*	%	*n*	%	
Vaccine-preventable diseases	91	71.7%	170	67.5%	93	63.7%	0.15
Effectiveness of vaccine	92	72.4%	157	62.3%	100	68.5%	0.25
Vaccine safety	75	59.1%	114	45.2%	80	54.8%	0.04 *
Vaccine adverse events/adverse reactions	84	66.1%	138	54.8%	87	59.6%	0.29
Contraindications to vaccine	91	71.7%	139	55.2%	80	54.8%	<0.001 *
Infant vaccination schedule	91	71.7%	160	63.5%	79	54.1%	0.05
Vaccination schedule for adults	80	63.0%	109	43.3%	63	43.2%	0.004 *
Communication techniques (risk–benefit communication)	68	53.5%	110	43.7%	59	40.4%	0.07
Communication methods for vaccine hesitancy	60	47.2%	94	37.3%	37	25.3%	0.005 *
Vaccine clinical trials and approval process	77	60.6%	79	31.3%	71	48.6%	<0.001 *

* *p*-value < 0.05.

**Table 4:** Comparison of immunization knowledge scores among healthcare profession students.

	Range	Medical Students			Nursing Students			Pharmacy Students			*p*-Value
		Mean	(SD)	Percentage of Average Correct Answers	Mean	(SD)	Percentage of Average Correct Answers	Mean	(SD)	Percentage of Average Correct Answers	
Healthcare professionals on recommended vaccines for administration	(0–15)	9.8	(2.4)	(57.8)	9.9	(2.1)	(58.3)	9.5	(2.1)	(55.6)	0.111
Voluntary vaccination	(0–17)	11.7	(2.3)	(68.8)	11.4	(1.6)	(67.2)	11.8	(1.8)	(69.6)	0.094
Vaccines administered at 2 months of age	(0–15)	10.6	(2.1)	(70.6)	9.8	(1.8)	(65.5)	9.8	(1.9)	(65.5)	<0.001 *
Vaccine-preventable diseases (VPDs)	(0–4)	2.4	(1.4)	(59.5)	1.6	(1.2)	(39.4)	2.1	(1.4)	(52.1)	<0.001 *
Basic knowledge about vaccination	(0–18)	8.8	(3.9)	(48.7)	7.0	(3.8)	(38.8)	7.9	(3.5)	(44.1)	<0.001 *
Total	(0–69)	43.3	(8.4)	(62.7)	39.7	(6.4)	(57.6)	41.2	(6.2)	(59.6)	<0.001 *

* *p*-value < 0.05.

## Data Availability

The datasets generated and/or analyzed in the current study are not publicly available due to ethical restrictions but are available from the corresponding author on reasonable request.

## Supplementary Materials

The following supporting information can be downloaded at: https://www.mdpi.com/article/10.3390/vaccines12121310/s1, Table S1-1: Knowledge of healthcare professionals on recommended vaccines for administration; Table S1-2. Knowledge of voluntary vaccination; Table S1-3. Knowledge of vaccines administered at 2 months of age; Table S1-4. Knowledge of vaccine-preventable diseases (VPDs); Table S1-5. Basic knowledge about vaccination; Table S2. Desire for improvement in vaccination education among healthcare students.
