# Supplementary material for: Vaccination Education Deficits and Vaccine Hesitancy Among Healthcare Students in Japan: A Cross-Sectional Study
Source: Vaccines (Basel). 2024 Nov 22;12(12):1310. doi: 10.3390/vaccines12121310 (PMC11680435; doi:10.3390/vaccines12121310)
Supplement: Supplementary file 1 [file vaccines-12-01310-s001.zip › vaccines-3291102-supplementary.pdf]

Supplementary Table S1

Table S1-1. Knowledge of healthcare professionals on recommended vaccines for administration

	Which of the following vaccines do you need to be vaccinated against as a health care professional? (True or False)
1	Four-component combination vaccine
2	Hepatitis B vaccine
3	Pneumococcal vaccine
4	Rubella vaccine
5	Rotavirus vaccine
6	Pertussis vaccine
7	Influenza vaccine
8	Hemophilus influenzae type b (Hib) vaccine
9	Measles vaccine
10	BCG vaccine
11	Meningococcal vaccine
12	COVID-19 vaccine
13	Varicella (chickenpox) vaccine
14	HPV vaccine
15	Japanese encephalitis vaccine

Table S1-2. Knowledge of voluntary vaccination

	Which are the voluntary vaccines? (True or False questions)
1	Four-component combination vaccine
2	Hepatitis B vaccine
3	Pneumococcal vaccine
4	Rubella vaccine
5	Rotavirus vaccine
6	Pertussis vaccine
7	Influenza vaccine
8	Hemophilus influenzae type b (Hib) vaccine
9	Measles vaccine
10	BCG vaccine
11	Meningococcal vaccine
12	COVID-19 vaccine
13	Varicella (chickenpox) vaccine
14	Human papilloma virus vaccine
15	Japanese encephalitis vaccine
16	Triple vaccine
17	Hepatitis A vaccine

Table S1-3. Knowledge of vaccines administered at 2 months of age

	Which of the following vaccines are administered at 2 months of age according to the standard schedule in Japan? (True or False questions)
1	Four-component combination vaccine
2	Hepatitis B vaccine
3	Pneumococcal vaccine
4	Rubella vaccine
5	Rotavirus vaccine
6	Pertussis vaccine
7	Influenza vaccine
8	Hemophilus influenzae type b (Hib) vaccine
9	Measles vaccine
10	BCG vaccine
11	Meningococcal vaccine

12	COVID-19 vaccine
13	Varicella (chickenpox) vaccine
14	Human papilloma virus vaccine
15	Japanese encephalitis vaccine

Table S1-4. Knowledge of vaccine-preventable diseases (VPDs)

Which of the following vaccines prevents bacterial meningitis?
Mumps vaccine
Hib vaccine
Varicella vaccine
Japanese encephalitis vaccine
**Which of the following vaccines prevents pertussis?**
Childhood pneumococcal vaccine
Mumps vaccine
Rubella vaccine
Quadruple vaccine (DTaP-IPV)
Measles rubella vaccine
Dual combination (DT)
**Which of the following vaccines prevent liver cancer?**
Hib vaccine
Hepatitis B Vaccine
Hepatitis A Vaccine
Rotavirus vaccine
**Which of the following best describes the rotavirus vaccine?**
Prevent encephalitis
Prevent cirrhosis and liver cancer
Prevent bacterial meningitis
Prevent infections that cause diarrhea

Table S1-5. Basic knowledge about vaccination

	Please select whether the content is correct or incorrect
1	If accompanied by symptoms such as mild coughing or a runny nose, it is advisable to refrain from vaccination.
2	Administering multiple vaccines simultaneously can place excessive burden on the body's immune system.
3	Varicella vaccine, when administered within 3 to 5 days after exposure to chickenpox, can prevent the disease or reduce its severity.
4	Children who are taking antibiotics for otitis media can still receive routine vaccinations.
5	The quadrivalent vaccine may have the potential to cause sudden infant death syndrome (SIDS).
6	The association between chronic conditions such as autism and multiple sclerosis and vaccine administration is supported by the latest scientific data.
7	Prior to vaccination, it is necessary to undergo a physical examination and medical interview conducted by a physician.
8	Most vaccines for children are administered intramuscularly.
9	There are no orally administered vaccines for children.
10	Inactivated vaccines require fewer doses compared to live vaccines.
11	It is desirable for each vaccine to be completed before the susceptible period.
12	Many live vaccines for children are typically administered starting at 6 months of age.
13	Vaccination against pertussis in children is an optional vaccine that incurs a cost.
14	Vaccination can prevent severe illness and complications.
15	Natural infection of pertussis does not leave severe sequelae.
16	Natural infection is safer for building immunity compared to vaccination.
17	Vaccines categorized as voluntary vaccines are mandated by law and administered at public expense (generally free), while vaccines for routine immunization are received at the individual's expense.
18	Administering multiple vaccines simultaneously is safe.

Supplementary Table S2

Table S2. Immunization course subjects among healthcare professional schools (multiple answers)

	Total		Medical students		Nursing students		Pharmacy students	
	n	%	n	%	N	%	n	%
Overall	525	100.0	127	100.0	252	100.0	146	100.0
Immunity	92	17.5	37	29.1	20	7.9	35	24.0
Infection	81	15.4	44	34.6	19	7.5	18	12.3
Public health	71	13.5	22	17.3	35	13.9	14	9.6
Pharmacology, drugs, and medicines	66	12.6	5	3.9	27	10.7	34	23.3
Pediatric nursing	49	9.3	1	0.8	48	19.0	-	-
Pediatrics	45	8.6	26	20.5	19	7.5	-	-
Microbiology	44	8.4	14	11.0	10	4.0	20	13.7
Hygiene	35	6.7	-	-	1	0.4	34	23.3
Maternal nursing	31	5.9	-	-	31	12.3	-	-
Pathology	23	4.4	4	3.2	9	3.6	10	6.8
Basic nursing science and nursing techniques	23	4.4	-	-	23	9.1	-	-
Practical training/exercises	19	3.6	4	3.2	8	3.2	7	4.8
Bacteria & viruses	18	3.4	13	10.2	3	1.2	2	1.4
Biology	17	3.2	4	3.2	4	1.6	9	6.2
Adult nursing	16	3.0	-	-	16	6.3	-	-
Infection nursing	14	2.7	-	-	14	5.6	-	-
Epidemiology and statistics	13	2.5	1	0.8	11	4.4	1	0.7
Obstetrics and gynecology	13	2.5	12	9.4	1	0.4	-	-
Public health nursing	13	2.5	-	-	13	5.2	-	-
Biochemistry	12	2.3	2	1.6	6	2.4	4	2.7
Introduction/fundamentals/overview	12	2.3	6	4.7	5	2.0	1	0.7
Prevention	11	2.1	3	2.4	6	2.4	2	1.4
Others	143	27.2	55	43.3	67	26.6	21	14.4
No/I do not know	144	27.4	29	22.8	77	30.6	38	26.0

Supplementary Table S3

Table S3. Desire for improvement in vaccination education among healthcare students

		Number of recorded units							
		All		Medical students		Nursing students		Pharmacy students	
		N	%	N	%	N	%	N	%
Category name	Category name	525	100.0	127	100.0	252	100.0	146	100.0
Acquiring comprehensive knowledge	Want to learn more about advantages and disadvantages	36	6.9	5	3.9	26	10.3	5	3.4
	Want to learn more about adverse reactions	32	6.1	7	5.5	16	6.3	9	6.2
	Want to learn more about sequelae and risks	13	2.5	3	2.4	6	2.4	4	2.7
	Want to learn more about the mechanism of action	10	1.9	3	2.4	3	1.2	4	2.7
	Necessity of vaccination	6	1.1	2	1.6	4	1.6	-	-
	Effectiveness of vaccines	9	1.7	1	0.8	7	2.8	1	0.7
	Increase lectures	24	4.6	7	5.5	9	3.6	8	5.5
	Want to hear more detailed and careful explanations	44	8.4	13	10.2	19	7.5	12	8.2
	Want to learn the contents in an easy-to-understand way	17	3.2	2	1.6	11	4.4	4	2.7
	Want to acquire knowledge	10	1.9	2	1.6	8	3.2	-	-
	Want to learn from pharmacy and pharmacist's point of view	5	1.0	-	-	-	-	5	3.4
Gaining communication skills	Can teach correct knowledge	7	1.3	3	2.4	3	1.2	1	0.7
	Perform role-playing.	15	2.9	2	1.6	8	3.2	5	3.4
	Practice how to explain	13	2.5	5	3.9	3	1.2	5	3.4
	Want to learn how to deal with people who are refusing	11	2.1	3	2.4	8	3.2	-	-
	Want to learn expertise and ability to communicate	12	2.3	2	1.6	4	1.6	6	4.1
	Can manage immunization schedule	5	1.0	-	-	4	1.6	1	0.7
Content aligned with practical application	Want to learn how to deal with patients	14	2.7	7	5.5	4	1.6	3	2.1
	Increase practical content	33	6.3	8	6.3	14	5.6	11	7.5
	Want to learn more specific topics	19	3.6	5	3.9	7	2.8	7	4.8
	Want to learn practical content	6	1.1	2	1.6	1	0.4	3	2.1
	Want to learn content aligned with clinical practice	14	2.7	8	6.3	4	1.6	2	1.4
	Can observe the field	11	2.1	1	0.8	2	0.8	8	5.5
Motivation for learning	Use the latest information as much as possible	7	1.3	5	3.9	2	0.8	-	-
	Encourage independent thinking and stimulate interest	14	2.7	4	3.2	6	2.4	4	2.7
	Encourage independent thinking and stimulate interest	9	1.7	1	0.8	5	2.0	3	2.1
	Not to memorize	3	0.6	-	-	2	0.8	1	0.7
	Prepare for national examinations	2	0.4	-	-	1	0.4	1	0.7
